# Non-target impact of fungicide tetraconazole on microbial communities in soils with different agricultural management

**DOI:** 10.1007/s10646-016-1661-7

**Published:** 2016-04-22

**Authors:** Sławomir Sułowicz, Mariusz Cycoń, Zofia Piotrowska-Seget

**Affiliations:** Department of Microbiology, University of Silesia, Jagiellońska 28, 40-032 Katowice, Poland; Department of Microbiology and Virology, School of Pharmacy with the Division of Laboratory Medicine, Medical University of Silesia, Jagiellońska 4, 41-200 Sosnowiec, Poland

**Keywords:** Tetraconazole, Orchard, Grassland, DGGE, PLFA, Biolog

## Abstract

Effect of the fungicide tetraconazole on microbial community in silt loam soils from orchard with long history of triazole application and from grassland with no known history of fungicide usage was investigated. Triazole tetraconazole that had never been used on these soils before was applied at the field rate and at tenfold the FR. Response of microbial communities to tetraconazole was investigated during 28-day laboratory experiment by determination of changes in their biomass and structure (phospholipid fatty acids method—PLFA), activity (fluorescein diacetate hydrolysis—FDA) as well as changes in genetic (DGGE) and functional (Biolog) diversity. Obtained results indicated that the response of soil microorganisms to tetraconazole depended on the management of the soils. DGGE patterns revealed that both dosages of fungicide affected the structure of bacterial community and the impact on genetic diversity and richness was more prominent in orchard soil. Values of stress indices—the saturated/monounsaturated PLFAs ratio and the cyclo/monounsaturated precursors ratio, were almost twice as high and the Gram-negative/Gram-positive ratio was significantly lower in the orchard soil compared with the grassland soil. Results of principal component analysis of PLFA and Biolog profiles revealed significant impact of tetraconazole in orchard soil on day 28, whereas changes in these profiles obtained for grassland soil were insignificant or transient. Obtained results indicated that orchards soil seems to be more vulnerable to tetraconazole application compared to grassland soil. History of pesticide application and agricultural management should be taken into account in assessing of environmental impact of studied pesticides.

**Electronic supplementary material:**

The online version of this article (doi:10.1007/s10646-016-1661-7) contains supplementary material, which is available to authorized users.

## Introduction

Orchards are specific environments especially subjected to the successive annual application of fungicides. Many reports have shown that fungicides application may affect the biomass of non-target microorganisms (Pal et al. 2008; Tejada et al. 2011), change their biochemical activity (Bending et al. 2007; Wang et al. 2009a, 2016; Milenkovski et al. 2010; Muñoz-Leoz et al. 2011) as well as structural (Wang et al. 2009b; Cordero-Bueso et al. 2014; You et al. 2016), functional (Muñoz-Leoz et al. 2011; Wang et al. 2012; Wu et al. 2015) and genetic diversity (Yen et al. 2009; Gu et al. 2010). Changes in microbial biota may be reflected in soil diversity, a key factor for the supplying of ecosystem goods and services to human society, which determines the ecological responses of terrestrial ecosystems to environmental change (Bardgett and van der Putten 2014). Therefore component of the intensive agriculture management, such as pesticide application, may influence the response of microorganisms to applied fungicides (Barrios 2007).

One of the most important fungicides abundantly used in the management practice of orchards are the demethylation inhibitors (DMIs) such as triazoles (Verweij et al. 2009). These fungicides inhibit the cytochrome P450 14-α sterol demethylase and stop the synthesis of fungal ergosterol (Amer et al. 2007). Multiple application of an azole fungicide alters fungal populations and in response to reducing sensitivity to DMIs new fungicides have to be applied (Holb and Schnabel 2007; Verweij et al. 2009). One of the compounds which is used to replace ineffective fungicides is a next generation DMI fungicide—triazole tetraconazole. The impact of tetraconazole on the soil environment is poorly studied and these data are mainly provided by the producers of the fungicide and two field study (Zhang et al. 2014a; Sułowicz and Piotrowska-Seget 2016). Microbial degradation, hydrolysis and photolysis proceed slowly in soil. Laboratory and field studies estimate the half-time (*T*_1/2_) dependent on its application concentration and soil texture in the range from 67 to 69 days (Alam et al. 2013; Zhang et al. 2014a) to over 1688 days (EPA 2005; EFSA 2008). Persistence of tetraconazole indicates a tendency to accumulate in the soil with successive annual applications. Therefore, a decision of the EU authorises limited use of tetraconazole for crops on the same field every third year (Council Directive 2009).

The objective of this study was to investigate the response of microbial communities from two silt loam soils with different history of soil management to triazole tetraconazole that had never been used on these soils before. Effect of this fungicide on microbial community structure, functional and genetic diversity as well as the enzyme activity was established in orchard, on which triazoles had been applied for many years and in grassland soil with no known history of fungicide application.

## Methods

### Soils characterization

In this study two soils with different history of management and pesticide application were used. Soil samples were collected from the surface layer (0–10 cm) from an apple orchard (Radzionków, Upper Silesia, Poland) in which triazoles but not tetraconazole had been systematically applied for the last 40 years and from an adjacent non-cultivated area with no known history of pesticide application. The collected soils were homogenised, freshly sieved with 2-mm mesh and stored for equilibrating purpose in field moist for 2 weeks at 4 °C prior to the analysis. Before mesocosm experiment soil was preincubated at 20 ± 2 °C for 5 days. According to the FAO system (FAO 2006) the soil was classified as Leptic Podzols. Based on a texture analysis (ISO 11277 2009), the soils were found to be silt loam (4 % clay, 59 % silt, 37 % sand and 7 % clay, 64 % silt, 29 % sand for orchard and grassland, respectively). The main soil characteristics were established: pH value of the aqueous soil extracts (1:2.5, w/v) (ISO 10390 2005), the organic matter (OM) content using dichromate oxidation in the presence of concentrated sulphuric acid (ISO 14235 1998) and the total nitrogen (N_tot_) content (ISO 11261 1995). Main characteristics for orchard soil were the following: pH, 4.58; OM, 1.69 %; and N_tot_, 0.13 %. The soil collected from the grassland characterised with following parameters: pH, 6.84; OM, 2.32 %; and N_tot_, 0.11 %.

### Mesocosm experiment

For each soil—orchard and grassland, respectively, three sets of three replicated mesocosms (2 kg) was prepared. The following treatments were studied: control and treatment with fungicide at the field rate (FR) and tenfold field rate (10FR). Tetraconazole was applied as an aqueous solution of the commercial formulation Domark 100EC (Isagro S.p.A, Italy) and the rates of fungicide corresponded to 0.1 and 1 mg of tetraconazole per kg of soil, respectively, assuming a homogeneous distribution of the fungicide to a depth of 10 cm and a soil density of 1.5 g cm^3^. Soil moisture content was adjusted to 40 % water holding capacity (WHC) and was held constant during experimental period. Mesocosms were incubated in the dark at 20 ± 2 °C for 28 days. Soil cores were periodically removed with a auger (2 cm diameter) for a determination of the microbial biomass and activity (on days 1, 7, 14, 21 and 28) and microbial structure and community level physiological profiles (1 and 28 days).

### Analysis of the soil microbial community structure by PCR-DGGE method

Total DNA extracted from soil samples (the GeneMATRIX Soil DNA Purification Kit, Eur_x_, Poland) was subjected to electrophoresis in 1.0 % (w/v) agarose gel and quantified by the spectrophotometer method (Biophotomether, Eppendorf). Primers F338 (5′-ACT CCT ACG GGA GGC AGC AG-3′) and R518 (5′-ATT ACC GCG GCT GCT GG-3′) were used for amplification of a fragment of the V3 region of the bacterial 16S RNA gene. Forward primers contained a 40-bp GC-clamp (5′-CGC CCG CCG CGC GCG GCG GGC GGG GCG GGG GCA CGG GGG G-3′) attached to the 5′ end (Muyzer et al. 1993). All PCR mixture contained following concentrations of 1× GoTaq Flexi Buffer (Promega), 2 mM MgCl_2_, 0.2 mM dNTP Mix (Promega), 0.5 µM of each primer (Sigma-Aldrich), 0.2 µg of DNA, and 1.5 U/µl GoTaq DNA Polymerase (Promega). PTC-118 Thermal Cycler (BIO-RAD, CA, USA) was used for DNA amplification with following steps: (i) an initial denaturation step of 95 °C for 10 min, (ii) 30 cycles of denaturation, annealing and extension (95 °C for 1 min followed by 53 °C for 1 min, with an extension step at 72 °C for 2 min), and (iii) a final extension at 72 °C for 12 min. The QIAquick PCR Purification Kit (Qiagen, USA) was used for purification of the PCR products.

The PCR products were analyzed in a 8 % (w/v) polyacrylamide gel (37.5:1 acrylamide:bis-acrylamide) composed of a linear denaturing gradient ranging from 40 to 70 %. Denaturant solutions were prepared by mixing the appropriate volumes of two (0–100 %) denaturant stock solutions (7 mol/l urea, and 40 % v/v formamide). Gels were run in a 1xTAE buffer with a constant voltage of 80 V for 14 h at 60 °C using a DCode Mutation Detection System (Bio-Rad, USA). Gels were stained with ethidium bromide (0.5 mg/mL), photographed and analyzed.

For calculation of the similarity values of the bacterial community based on the DGGE profiles Quantity One^®^ Software (Bio-Rad, USA) was used. The phylogenic dendrograms were constructed on the basis of the band presence/absence and band weighting (band density) by applying the Dice coefficient and the unweighted pair-group method with the use of arithmetic averages (UPGMA). The Shannon index (*H*) was calculated according to the equations *H* = −∑*pi*(ln *p*_*i*_), where *p*_*i*_ is the ratio between the specific band intensity and the total intensity of all bands; species richness (S) values were estimated as the total number of bands in each sample, while evenness index *E* = *H/H*_*max*_ = *H/*ln*S* (Cycoń et al. 2013).

### Biomass and the structure of microbial communities based on the PLFA approach

The biomass of distinct microbial groups and the community structure of the soil microorganisms was determined using the phospholipid fatty acids (PLFA) approach. PLFAs were extracted as described by Frostegard et al. (1993) with minor modifications. Briefly, the lipids from 2 g of fresh soil, extracted with a chloroform:methanol:citric buffer mixture (1:2:0.8 v/v/v), were fractionated on silicic acid columns (Supelco). The fraction of phospholipids was subjected to mild alkaline methanolysis. The fatty acid methyl esters were separated using a gas chromatograph (Hewlett-Packard 6890, USA) on a HP-Ultra 2 capillary column (cross-linked 5 % phenyl-methyl silicone; 25 m, 0.20 mm ID, film thickness 0.33 μm) with hydrogen as the carrier gas. The PLFAs were detected using a flame ionisation detector (FID) and identified using the MIDI Microbial Identification System Software (Sherlock TSBA40 method and TSBA40 library; MIDI Inc., Newark, DE, USA). For the quantitative determination of fatty acids nonadecanoic acid (19:0) as an internal standard was used. Mass of PLFAs was expressed as nanomoles per gram of dry soil. The analysis was based on marker fatty acids characteristics for Gram-negative bacteria (16:1ω7c, cy17:0, 18:1ω7c, cy19:0), Gram-positive bacteria (i15:0, a15:0, i16:0, i17:0, a17:0) and actinomycetes (10Me16:0, 10Me17:0, 10Me18:0) (Moore-Kucera and Dick 2008). The sum of mass of these fatty acids referred the bacterial biomass, where as all isolated PLFAs in the range of 9:0–20:0 carbon atoms were considered as the total PLFAs mass. The 18:2ω6.9c and 18:1ω9c biomarkers were used to calculate the fungal PLFAs (Frostegård et al. 2011). Microbial stress indices, such as the Gram-negative/Gram-positive (GN/GP) (Zhang et al. 2014b), the fungi/bacteria PLFAs ratio (F/B), the cyclo/monounsaturated precursor (cy17:0 + cy19:0/16:1ω7c + 18:1ω7c) PLFAs ratio, the saturated/monounsaturated (S/M) PLFAs ratio (Moore-Kucera and Dick 2008), were calculated to describe the stress level caused by various management techniques and tetraconazole application on the microbial communities.

Additionally, the changes in the structure of soil microbial communities in response to the addition of tetraconazole were determined by analysis of the mass of fatty acids biomarker in PLFA profiles (nmol/g g dry soil). Results were analysed at the beginning and at the end of the experiments by a principal component analysis.

### Community-level physiological profile (CLPP) analysis

Biolog method and EcoPlates™ (Biolog Inc., CA, USA) were used to establish the changes in the CLPPs of the soil microbial communities at the beginning (day 1) and end (day 28) of the experiment. Each 96-well plate was inoculated with 125-µl aliquots from 10^−2^ soil suspensions (in a 0.85 % NaCl solution). The colour development in each well was recorded as the optical density (OD) at 590 nm using a Perkin Elmer Victor™ X5 spectrophotometer at regular 12-h intervals for 5 days. A raw OD data for each well was blanked against the control well. The profile of substrates utilization was assessed through the area under the absorbance versus time curve (AUC) standardized by the average area under the curve calculated for each substrate (Clivot et al. 2012; Montes-Borrego et al. 2013). Changes in the Biolog profiles were established by the principal component analysis. Additionally, the Shannon index (biodiversity index) was calculated according to the following equation: *H* = −∑ *p*_*i*_(ln *p*_*i*_), where *p*_*i*_ is the proportion of the corrected absorbance value of each well to that of all of the wells. Substrate richness (*Rs*) was described as the number of utilized substrates. McIntosh index (*E*) described the evenness or homogeneity of substrate utilization (Zhang et al. 2014b).

### Fluorescein diacetate hydrolysing activity (FDA)

FDA hydrolysing activity, which reflects the total soil microbial activity, was measured according to Adam and Duncan (2001). Briefly, for each of the soils tested triplicate 2-g subsamples were incubated at 30 °C for 20 min on a rotary shaker with 15 mL of a 60 mM potassium phosphate buffer (pH 7.6) and 0.2 mL of FDA (1 mg/mL). Blanks were prepared by adding 0.2 mL of distilled water instead of the FDA. The hydrolysis was stopped by adding 15 mL of a chloroform/methanol (2:1 v/v) mixture. The suspension was subsequently centrifuged at 3000 rpm for 5 min The concentration of free fluorescein in the filtered solution was measured at 490 nm using a Thermo Spectronic Helios Epsilon spectrophotometer. Fluorescein was estimated in reference to the calibration curves and the results were calculated per gram of dry soil.

### Data analysis

The data on the microbial parameters from the orchard and grassland soil were tested with analysis of variance (ANOVA). Comparison of the orchard and grassland soils was estimated based on the values of microbial parameters from control samples by the two-way factorial ANOVA with soil and time as factors. Changes in the values of the microbial parameters during the experiment in response to fungicide treatments were assessed by two-way ANOVA (dose and time as factors) in each soil separately. Post-hoc comparisons (LSD tests) were performed to identify the important differences between fungicide treated and control soils at each sampling time at *P* < 0.05 when interaction of dose and time factors was statistically important or between means for dosages when only the effect of main factor was statistically important. The averages of triplicate data (n = 3) ± standard deviation (SD) are presented in the figures and tables. The averages of three CLPP or PLFA data points obtained from different treatments at the beginning and at the end of the experiment were analysed using principal component analysis (PCA) based on a correlation matrix. PCA-axes values were analysed by two-way multivariate analysis of variance (MANOVA) and post hoc LSD tests (*P* < 0.05) were used for the statistical testing of the separation of the profiles along each PC. The statistical analyses were performed by the Statistica 10.0 PL Software package.

## Results

### DGGE analysis

**Fig. 1:**
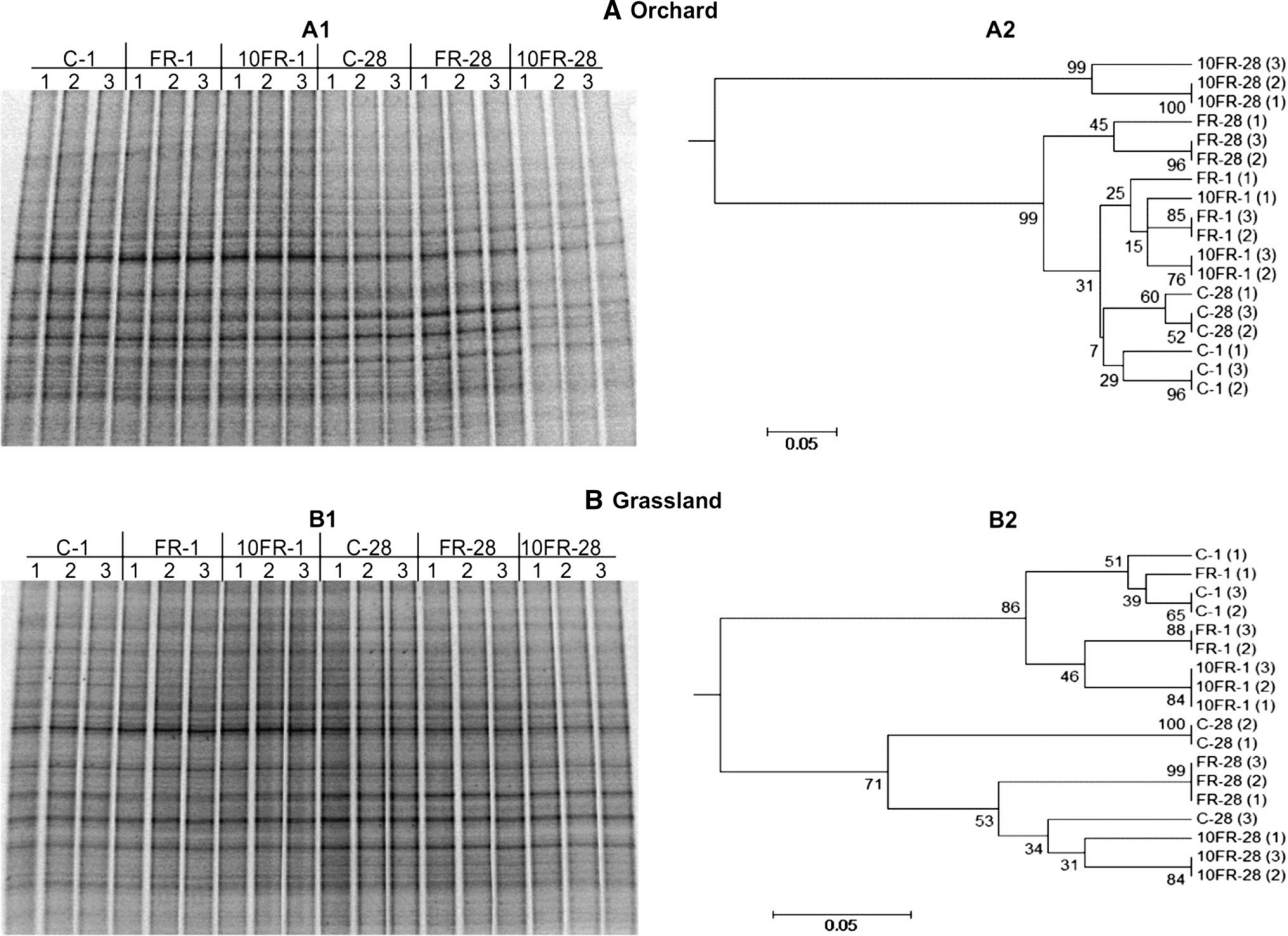
DGGE profiles (*A1* and *B1*) and phylogenic dendrogram (*A2* and *B2*) for PCR-amplified fragments of the 16S RNA gene for control (C), FR and 10FR treated orchard **(a)** and grassland **(b)** soils on days 1 and 28 following tetraconazole treatment

Analysis of DGGE patterns revealed that FR and 10FR tetraconazole affected the structure of the soil bacterial community during the 28-day experiment. DGGE profiles generated from the replicates of tetraconazole dosages and the control for orchard and grassland soils were very similar regarding complexity and band position (Fig. 1A1, B1). The cluster analysis of DGGE profiles obtained from orchard soil indicated that dosage of fungicide was the main factor responsible for profiles separation (Fig. 1A2). Control DGGE profiles from days 1 and 28 showed the highest similarity of band patterns, whereas the profiles from FR and expecially 10FR-treated soils 28 days after fungicide application varied the most in comparison with control profiles. Similarly, the analysis of the impact of tetraconazole on microbial structure in grassland soil also revealed that apart time, which was the most important factor grouping the DGGE profiles, funcigice dosage influenced the grouping of DGGE profiles within sampling days (Fig. 1B2). DGGE band presence/absence and band weighting (band density) analyses revealed that several bacterial community members, especially in orchard soil, were affected by the fungicide treatment (Fig. 1).

**Fig. 2:**
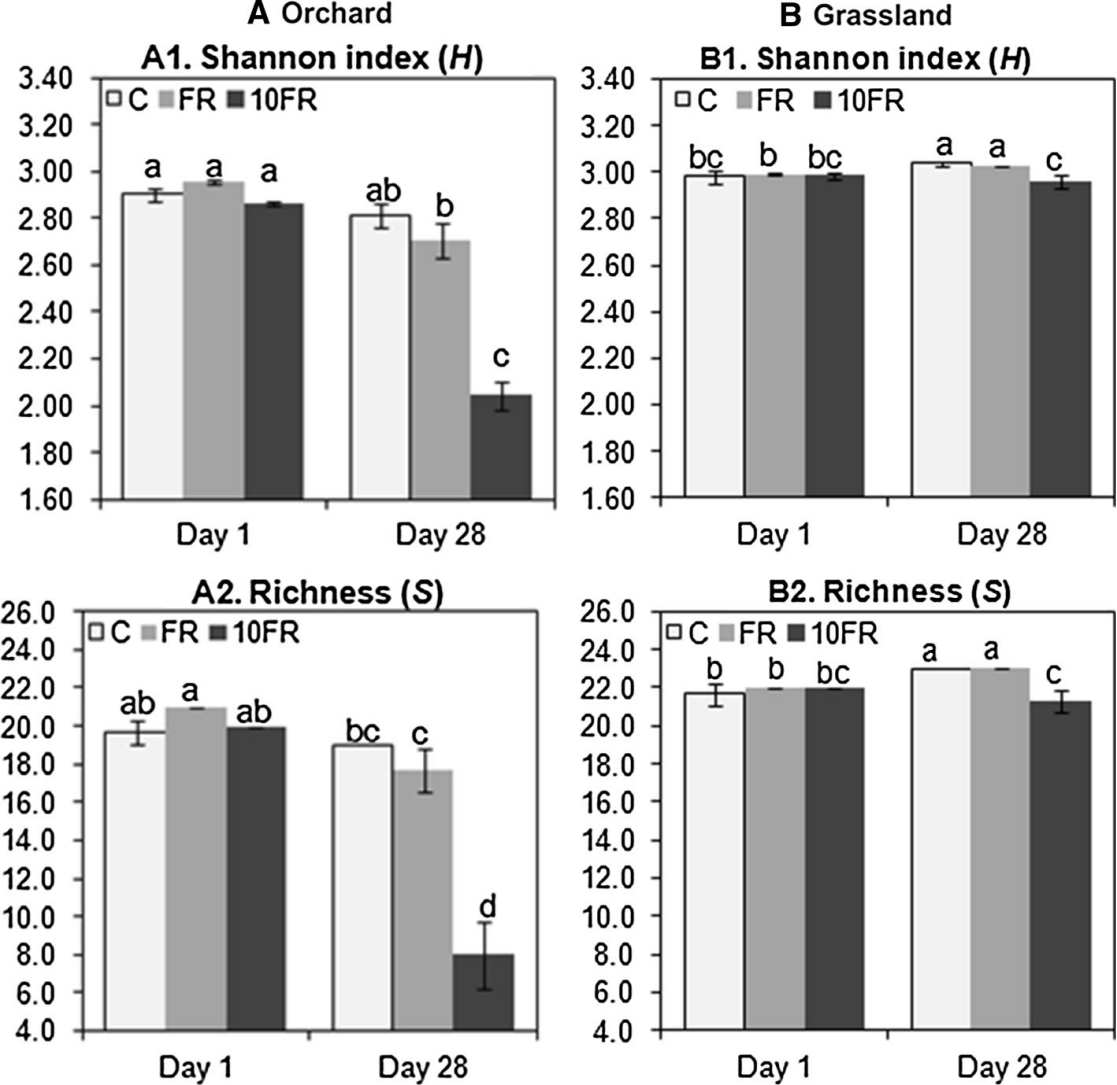
The genetic diversity indices for control (C), FR and 10FR treated orchard **(a)** and grassland **(b)** soils on days 1 and 28 following tetraconazole treatment. The data presented are the means and standard deviations of three replicates. *Different letters* (within each soil) indicate significant differences (*P* < 0.05, LSD test), considering the effects of the pesticide dosage and time

Comparison of the values of *H* and *S* indices calculated for orchard and grassland soils revealed significant (*P* < 0.001) differences between tested soils, and soil factor explained 74 and 85 % of observed variance in *H* and *Rs* indices, respectively (Table 1). Both indices obtained for orchard were significanlty lower in comparison with grassland soil. Moreover, response of the microbial communities to tetraconazole application differed depended on the soil management. The application of tetraconazole significantly affected the biodiversity index (*P* < 0.001 for orchard, *P* < 0.01 for grassland) and the richness (*S*) values (*P* < 0.001 for both soils) in orchard and grassland soils (Fig. 2). In both soils effects of tetraconazole treatment were not observed immediately after application of pesticide but on day 28. The impact of fungicide was higher in soil with long history of pesticide application, where *H* and *S* values (for 10FR-treated soil) significantly decreased about 30 and 58 %, respectively, in comparison with control (Fig. 2a). In grassland soil decrease of *H* values observed on day 28 in response to 10FR treatement was almost ten-times and eight-times smaller in comparison with orchard soil (Fig. 2b). Changes of evenness index *E*_*H*_ were not significantly affected (data not shown) during the experimental period.

**Table 1:** Comparison of orchard (O) and grassland (G) soil based on the values of microbial parameters

Method	Microbial parameter	Soil	Mean ± SD	*P* value			Variance explained by soil factor (%)
				Soil	Time	Soil × time	
DGGE	DGGE *H*	O	2.86 ± 0.06	**<0.001**	0.423	0.005	73.8
		G	3.01 ± 0.04				
	DGGE *S*	O	19.33 ± 0.52	**<0.001**	0.195	0.003	85.3
		G	22.33 ± 0.82				
	DGGE *E*	O	0.97 ± 0.01	0.619	0.056	0.177	1.7
		G	0.97 ± 0.00				
PLFA	Total PLFAs	O	82.6 ± 16.2	0.608	0.195	0.228	0.4
		G	81.0 ± 10.4				
	Bacterial PLFAs	O	36.06 ± 4.37	**0.019**	0.173	0.306	16.8
		G	40.03 ± 4.76				
	Gram-negative PLFAs	O	15.01 ± 1.76	**<0.001**	0.205	0.425	40.9
		G	18.37 ± 2.38				
	Gram-positive PLFAs	O	13.68 ± 1.98	0.490	0.173	0.418	1.6
		G	14.12 ± 1.64				
	Actinomycetal PLFAs	O	7.38 ± 1.07	0.650	0.238	0.051	0.6
		G	7.53 ± 1.12				
	Fungal PLFAs	O	7.25 ± 0.97	0.134	0.247	0.105	6.5
		G	7.75 ± 0.99				
	pre/cy ratio	O	0.61 ± 0.09	**<0.001**	<0.001	0.019	78.4
		G	0.37 ± 0.04				
	S/M ratio	O	1.08 ± 0.27	**<0.001**	<0.001	0.019	52.6
		G	0.68 ± 0.06				
	GN/GP ratio	O	1.11 ± 0.10	**<0.001**	0.665	0.224	51.0
		G	1.30 ± 0.10				
	F/B ratio	O	0.06 ± 0.01	0.635	0.928	0.145	0.8
		G	0.06 ± 0.01				
Biolog	Biolog *H*	O	2.99 ± 0.12	0.897	0.355	0.094	0.1
		G	2.98 ± 0.29				
	Biolog *Rs*	O	25.67 ± 2.50	0.740	0.206	0.073	0.8
		G	25.00 ± 5.06				
	Biolog *E*	O	0.92 ± 0.01	0.709	0.823	0.185	1.4
		G	0.93 ± 0.03				
FDA	FDA	O	1.71 ± 0.54	**<0.001**	<0.001	<0.001	13.7
		G	2.06 ± 0.35				

Significant values of soil factor for particular microbial parameters are bold

*DGGE H* the Shannon index *H*, *DGGE S* species richness *S*, *DGGE E* evenness index *E, S/M* the saturated/monounsaturated PLFAs ratio, *cy/pre* the cyclo/monounsaturated precursors ratio, *GN/GP* the Gram-negative/Gram-positive PLFAs ratio, *F/B* the fungi/bacteria PLFAs ratio, *Biolog H* the Shannon index (biodiversity index), *Biolog Rs* Substrate richness, *Biolog E* the evenness/homogeneity of substrate utilization, *FDA* Fluorescein diacetate hydrolysing activity. PLFA—nmol g^−1^ g dry soil, FDA—µg fluorescein g^−1^ dry soil

### Structural biodiversity of microbial communities based on the PLFA approach

**Fig. 3:**
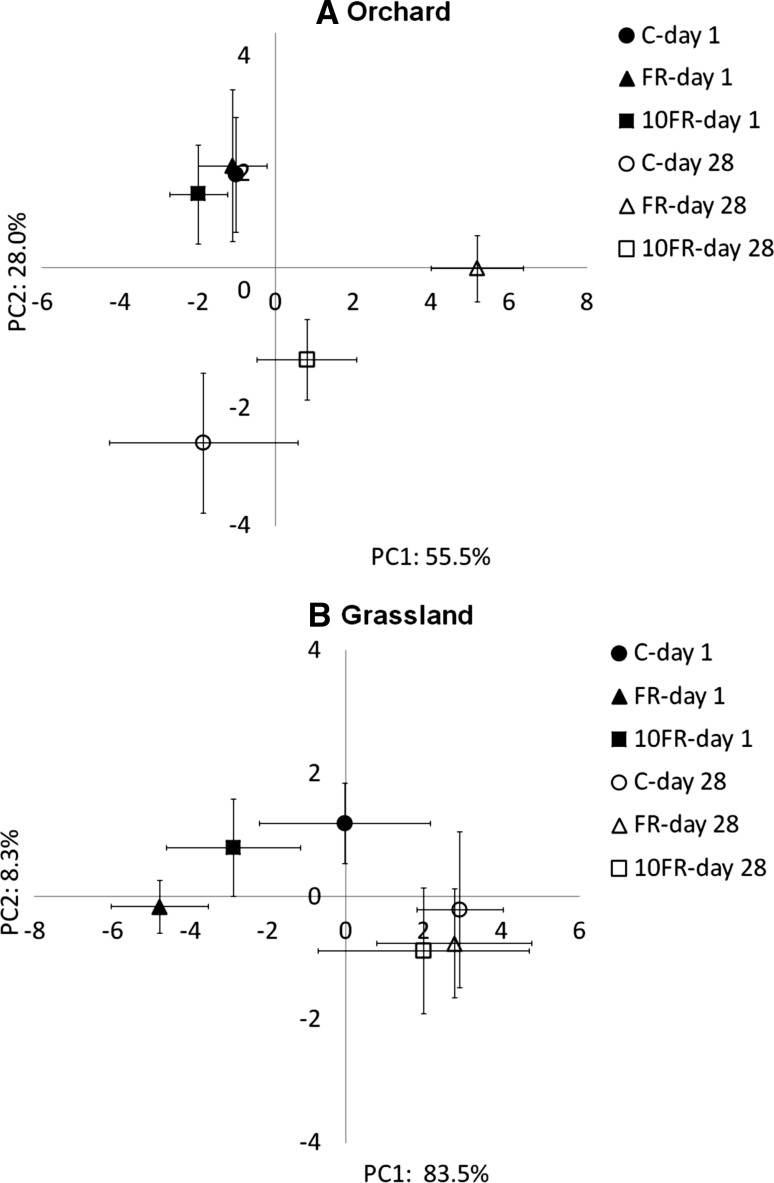
PCA plots of the PLFAs biomarkers profiles obtained for orchard **(a)** and grassland **(b)** soils treated with FR and 10FR of tetraconazole for one (*closed symbols*) and 28 days (*open symbols*); *C* control

The effect of tetraconazole on structural diversity was determined at the beginning and at the end of the experimental period. The structure of microbial communities was characterised through the principal component analysis (PCA) of the profiles of fatty acids biomarkers. PC1 and PC2 accounted for 55.5 and 28.0 % of the total variability in the orchard soil, respectively (Fig. 3a). A significant separation (MANOVA, *P* < 0.01) on the PC1 axis between the PLFA profiles were obtained from the control and the tetraconazole-treated orchard soils at the end of the experimental period. As shown in Table 2 fatty acids loadings on PC1 indicated that the mass of PLFA biomarkers at the end of experiment was higher in control profiles in comparison with tetraconazole-treated soils. Additional two-way ANOVA analysis of mass of individual biomarkers indicated impact of tested dosages of tetraconazole on the structure of microbial communities on day 28, when significant decrease (*P* < 0.05) in mass of three biomarkers of GP bacteria—i15:0, i16:0, a15:0 was observed.

**Table 2:** Phospholipid biomarker loadings on the PC1 and PC2 obtained from the analysis of the PLFA profiles

Biomarkers of PLFA	Orchard		Grassland	
	PC1	PC2	PC1	PC2
Gram-positive				
i15:0	**−0.95**	0.20	−**0.84**	−0.42
a15:0	−0.71	0.65	−**0.94**	0.07
i16:0	−**0.91**	−0.37	−**0.99**	−0.04
i17:0	−0.37	−0.63	−**0.77**	−0.58
a17:0	−0.47	0.52	−**0.91**	0.32
Gram-negative				
16:1ω7c	−0.71	0.59	−**0.98**	0.12
cy17:0	−**0.91**	−0.34	−**0.93**	−0.29
18:1ω7c	−**0.97**	0.11	−**0.98**	0.14
cy19:0	−0.59	−**0.79**	−**0.89**	−0.34
Actinomycetal				
10Me16:0	−**0.87**	0.31	−**0.95**	0.16
10Me17:0	−0.23	−0.74	−**0.89**	0.35
10Me18:0	−0.51	−**0.81**	−**0.96**	−0.09
Fungal				
18:2ω6.9c	−**0.76**	0.51	−0.73	0.38
18:1ω9c	−**0.95**	−0.15	−**0.98**	0.14

High correlation (r > 0.75) with PC axes of phospholipid biomarkers is bold

First two principal component explained 83.5 and 8.3 % of the total variability, respectively, in the grassland soil (Fig. 3b). PC1 distinguished profiles according to the time. All biomarkers showed high correlation (r > 0.75) with negative part of PC1, what indicated that the highest content of the biomarker fatty acids was observed in profiles from fungicide-treated soils obtained at the beginning of the experiment (Table 2). Slight separation between control and tetraconazole-treated soil on day 1 was observed, however significance of these changes were not confirmed by MANOVA analysis. Also statistical analysis of mass of fatty acids did not reveal significant changes in composition of individual biomarkers in response to fungicide application.

### Microbial biomass and stress indices

Total PLFAs mass, mass of GP, actinomycetal and fungal biomarkers did not significantly differ between orchard and grassland soil (Table 1). Only mass of GN PLFAs depended on the soil and was significantly (*P* < 0.001) higher in grassland soil in comparison with orchard, what resulted also in significantly higher mass of bacterial PLFAs in the first soil. In contrast to results obtained from PLFAs mass, history of management had significant impact on the values of ratios of microbial stress indices. The mean values of the ratio of cyclopropyl to monoenoic precursors (cy/pre) and saturated to monounsaturated (S/M) PLFAs calculated from the PLFAs collected from the orchard soil were significantly (*P* < 0.001) higher, and GN/GP ratio was significantly (*P* < 0.001) lower than those calculated for grassland soil. Soil factor explained 51–78 % of observed variance in the values of these stress ratio. Only the (F/B) ratio showed no statistically differences for both soil and was not depended on the soil factor.

An ANOVA analysis of mass of total PLFAs and individual groups of microbial markers obtained during the 28-day experiment generally showed no significant changes in response to fungicide application (Table S1). Effect of tetraconazole on soil fungi biomass was not observed in either of soils. A two-way ANOVA analysis of microbial stress indices—cy/pre, S/M, F/B and GN/GP did not reveal a significant distinct impact of tetraconazole on these parameters during the experimental period in either of the soils. Only small fluctuation as significant (*P* < 0.05) increase of GN/GP ratio in orchard soil and pre/cy ratio in grassland soil in response to tetraconazole application was observed (Table S1).

### Community-level physiological profile CLPP analysis

**Fig. 4:**
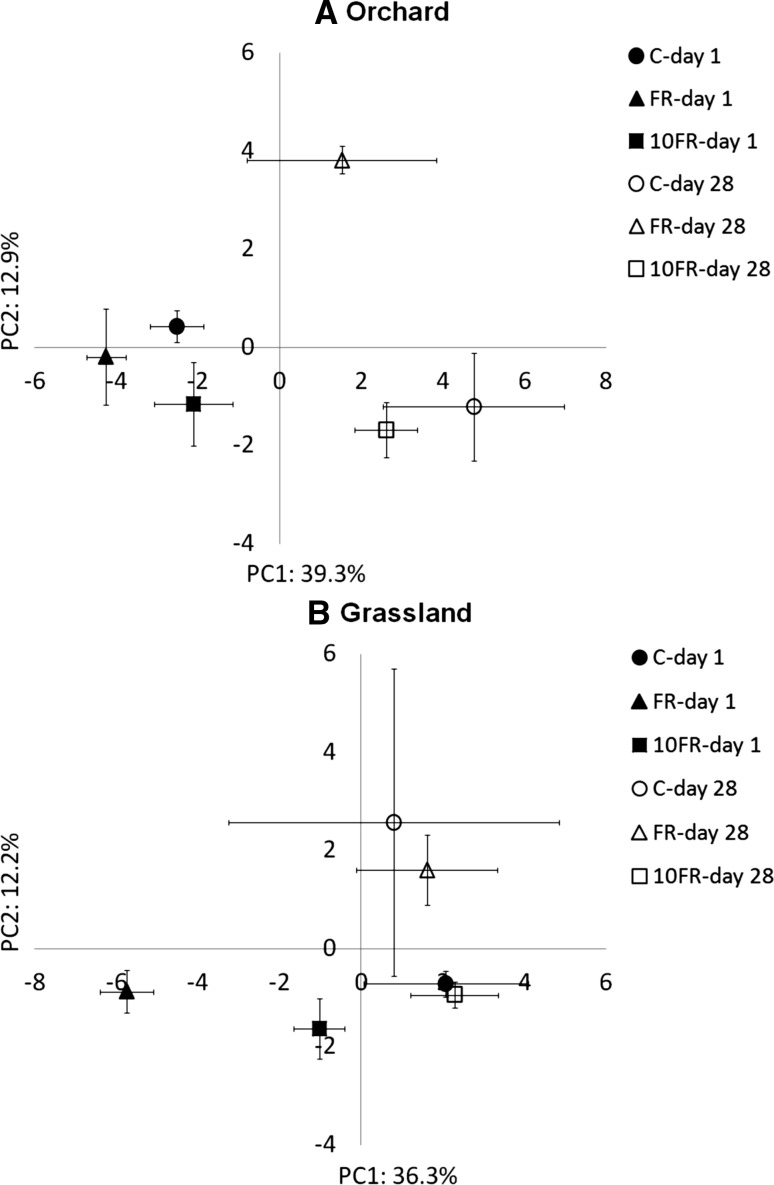
PCA plot of the CLPPs of the microbial communities in orchard **(a)** and grassland **(b)** soils that had been treated with FR and 10FR of tetraconazole for days 1 (*closed symbols*) and 28 (*open symbols*); *C* control

The effect of tetraconazole on the functional diversity of heterotrophic bacteria communities was determined by the Biolog Eco-Plate system. A principal component analysis of the CLPPs obtained for the orchard soil showed that PC1 and PC2 accounted for 39.3 and 12.9 % of the total variance in the data, respectively (Fig. 4a). PC1 distinguished (MANOVA, *P* < 0.001) the Biolog profiles according to time of soil sampling. An analysis of the substrate loadings on PC1 and PC2 showed that most of substrates were highly correlated with negative part of PC1, what indicated higher utilisation of substrates at the beginning of the experiment (Table S2). Additionally, the PCA results revealed differences in the metabolic potential of the microbial communities in the soils that had been treated with tetraconazole. On day 1 significant changes (*P* < 0.05) in Biolog profiles in response to 10FR tetraconazole were observed in comparison with the control soil along PC2. In turn 28 days later significant effect (PC2; *P* < 0.05) of the FR application of tetraconazole on the metabolic potential was observed. Microbial communities from FR soil characterized high ability to utilization of two carboxylic acids—2-hydroxy benzoic acid and α-ketobutyric acid (Table S2).

Principal component analysis of CLPPs obtained for the grassland soil revealed transient changes in the metabolic potential in response to tetraconazole application (Fig. 4b). Profiles from soil treated with FR tetraconazole significantly differed compared with the control on day 1 along PC1 (MANOVA, P < 0.05), which explained 36.3 % of the total variability. An application of FR of the fungicide significant increased the microbial potential for the utilisation of the substrates, what showed the substrate loadings on PC1 and PC2 (Table S2). The Biolog profiles obtained at the end of experimental time revealed that the utilisation of the substrates was generally lower in comparison with day 1 and no significant changes in response to the fungicide application were detected in comparison to untreated soil.

**Fig. 5:**
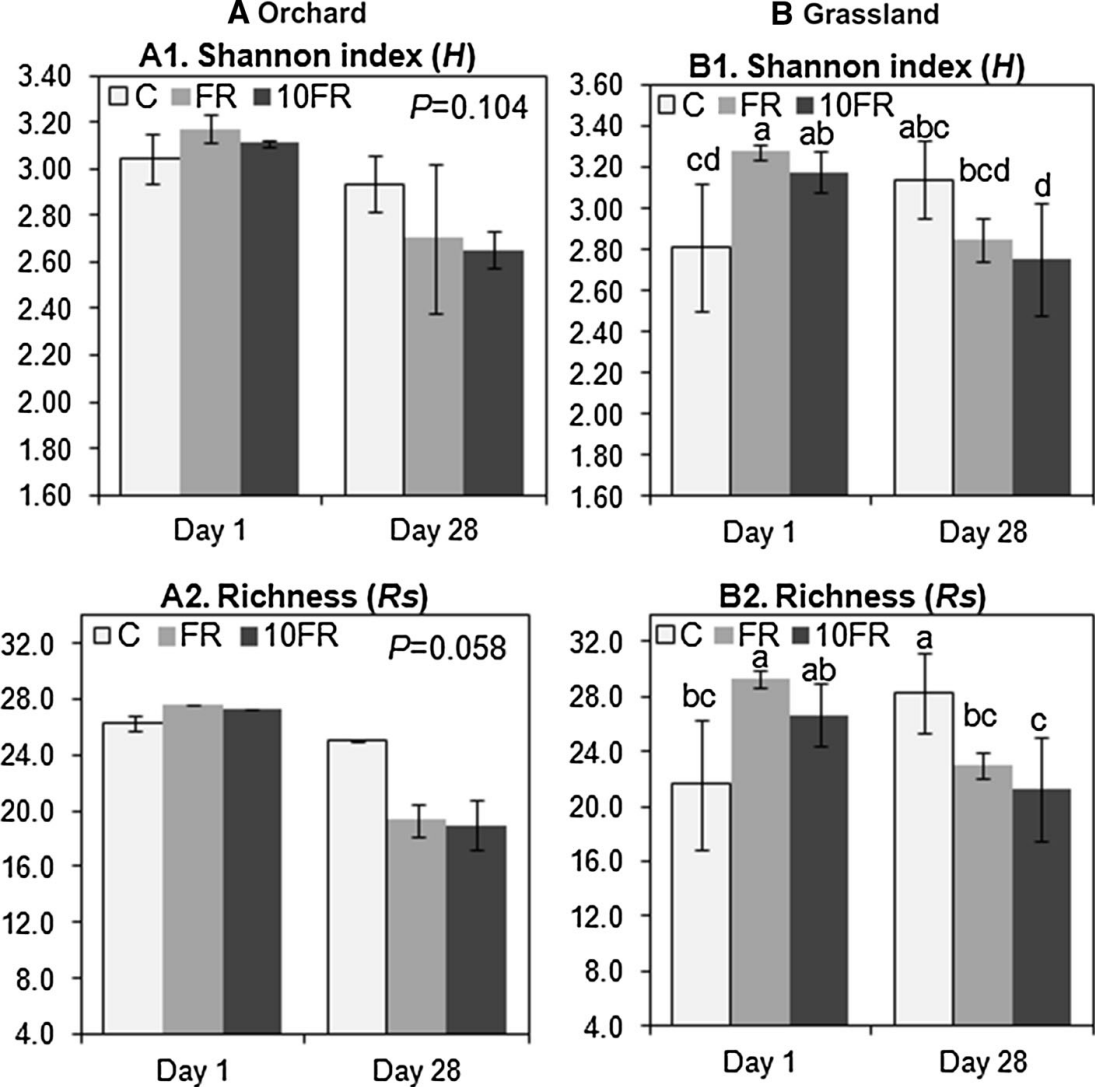
The functional diversity indices for control (C), FR and 10FR treated orchard **(a)** and grassland **(b)** soils on days 1 and 28 following tetraconazole treatment. The data presented are the means and standard deviations of three replicates. *Different letters* (within each soil) indicate significant differences (*P* < 0.05, LSD test), considering the effects of the pesticide dosage and time

Analysis of the changes in values of the Shannon index for the CLPPs in response to the tetraconazole application revealed significant impact of the fungicide on the functional biodiversity only in soil without previous history of pesticide application (Fig. 5). At the beginning significant (*P* < 0.05) increase of Shannon’s index was observed in soil treated with FR and 10FR. In contrast, on day 28 significant decrease of *H* values, compared with control, was observed in soil after 10FR application (Fig. 5B1). Observed changes in *H* values were connected with in the *Rs* values (Fig. 5B2). Changes of *E* were not significantly affected (data not shown) during the experimental period.

### Fluorescein diacetate hydrolysing activity (FDA)

The total microbial activity determined by FDA hydrolysis was significantly higher (*P* < 0.001) in grassland soil in comparison to orchard (Table 1). However, for both soils the two-way ANOVA analysis indicated no significant dose × time interaction and only main effect (*P* < 0.001) of tetraconazole application on total microbial activity in both soils was observed (Table 3). In the orchard soil increase in response to FR and 10FR tetraconazole has been observed. Contrary, in the grassland soil generally decrease in microbial activity in soil treated with 10FR has been detected.

**Table 3:** Total microbial activity for orchard (O) and grassland (G) soils after treatment at FR and 10FR of tetraconazole as estimated by the FDA reaction (µg fluorescein g^−1^ dry soil)

Soil	Pesticide treatment	Mean	Day				
			1	7	14	21	28
O	*P value*	<*0.001*	*0.058*				
	Control	1.71 ± 0.54^b^	1.58 ± 0.18	1.03 ± 0.15	2.52 ± 0.11	1.41 ± 0.03	1.99 ± 0.11
	FR	2.03 ± 0.55^a^	1.59 ± 0.48	1.43 ± 0.09	2.75 ± 0.15	1.97 ± 0.10	2.39 ± 0.09
	10FR	2.15 ± 0.58^a^	1.66 ± 0.34	1.53 ± 0.12	3.07 ± 0.17	2.25 ± 0.11	2.26 ± 0.06
G	*P value*	<*0.001*	*0.748*				
	Control	2.06 ± 0.34^a^	2.45 ± 0.11	1.84 ± 0.07	2.42 ± 0.21	1.85 ± 0.12	1.71 ± 0.15
	FR	1.97 ± 0.28^a^	2.28 ± 0.13	1.84 ± 0.02	2.21 ± 0.23	1.90 ± 0.05	1.63 ± 0.16
	10FR	1.85 ± 0.32^b^	2.19 ± 0.03	1.66 ± 0.14	2.20 ± 0.03	1.74 ± 0.18	1.46 ± 0.04

The data presented are the means and standard deviations of three replicates. Different letters indicate significant differences (*P* < 0.05, LSD test), considering the effects of the pesticide dosage for each soil

## Discussion

In this study the response of the microbial communities in soils with different management, including history of pesticide application to fungicide tetraconazole was estimated by DGGE, PLFA, Biolog and FDA methods. The EFSA report (Puglisi 2012) deals with the response of microorganisms to pesticides revealed that these methods are the most popular for assessment of the effect of pesticide and especially techniques analysing the structural and functional diversity of microbial communities are the most sensitive tools. These methods were also used for the differentiation of the soil by management practice (Shishido et al. 2008; Marshall et al. 2011; Montecchia et al. 2011; Montes-Borrego et al. 2013). In our study analysis of DGGE profiles showed that the history of soil management practices was reflected in the structure of bacterial communities. Furthermore, in contrast to other studies on fungicides, i.e. Bending et al. (2007), Tejada et al. (2011) or Thirup et al. (2001), tetraconazole application into the soils, which had never been sprayed with this fungicide, negatively affected soil bacterial biodiversity. As presented in DGGE dendrogram the effect of FR and 10FR tetraconazole on soil bacterial showed that main differences are between the treatments and the controls in orchard soil. It indicated that bacteria in this soil were more susceptible to applied fungicide in comparison with bacteria from grassland soil. The higher decrease of the values of *H* and *S* indices calculated for microbial communities from orchard soil in comparison with the values of these indices estimated for microbial populations from grassland soil confirmed this phenomenon. Higher susceptibility of microbial community from orchard soil may be explained by lower diversity of this community. The high sensitivity of even one group of bacteria to pesticide and its elimination from microbial community may result in significant decrease in total biodiversity. Similar, susceptibility of bacterial community to the fungicidal stress was observed in the study of carbendazim application (Tortella et al. 2013). Differences in response of microbial communities to applied fungicide may be connected with physico-chemical soil properties, i.e. pH or organic matter content. In our study orchard soil had lower pH and content of organic matter, however a decrease in the pH value of this soil may results from the management. It has been reported that long term use chemical fertilizers lowering the soil pH over time (Bradley et al. 2006) and extensive agriculture decrease the organic matter content (Tilman et al. 2002).

Differentiation of microbial response to applied tetraconazole depended on history of soil management was indicated also by the results obtained by PLFA. Only in the orchard soil tested dosages of tetraconazole caused changes in PLFA profiles, which were observed at the end of experimental period. Additionally, the application of fungicide tetraconazole in both dosages in this soil significantly decreased mass of the GP biomarkers, what resulted in increasing of the value of GN/GP ratio. These changes may indicate growth inhibition or the death of some GP species in response to tetraconazole application into silt loam soil. Decrease in the mass of microbial biomarkers, therein GP fatty acids, was also observed in other silt loam soils treated with tetraconazole (Zhang et al. 2014a) or fluopyram (Zhang et al. 2014b). In contrast, the increase in the mass of GP biomarkers was observed in a sandy loam and sandy clay loam soils in response to iprodione (Miñambres et al. 2010) and tetraconazole (Sułowicz and Piotrowska-Seget 2016) application as well as in sediments exposed to the fungicide captan (Widenfalk et al. 2008). It suggested that the soil texture modifies behaviour of the pesticides in the soil (Sanchez-Martin et al. 2006; Tejada 2009).

Surprisingly, in our study fungicide tetraconazole had shown no significant effect on biomass of soil fungi. Similarly, no effect of application of this fungicide on fungal community was observed in our earlier study in sandy clay loam (Sułowicz and Piotrowska-Seget 2016) and in response to benomyl (Marshall et al. 2011). In contrast, Zhang et al. (2014a) observed significant changes in fungal PLFAs mass in soil with no known history of pesticide application. In that study initial decrease in response to tetraconazole application has changed into significant increase since day 30. Contrary, results of other studies indicated that captan (Widenfalk et al. 2008) or fluopyram (Zhang et al. 2014b) significantly decreased the fungal biomass.

Significantly higher values of the pre/cy and S/M indices obtained from the PLFAs from the orchard soil indicated the presence of the stress condition in the orchard soil ecosystem which may be related with the agriculture management and successive annual application of pesticides. Also other studies revealed increase in the abundance of cyclopropyl fatty acids (Podio et al. 2008) and in the cy/pre PLFAs ratio (White et al. 2010) in the response to fungicide application.

The changes in the microbial community structure in orchard soil also corresponded with changes in the pattern of substrate utilization. Differences between PLFA profiles were the highest between control and FR-treated soils on day 28 and similarly significant impact of FR tetraconazole was determined for CLPP. This effect may resulted from the appearance of specific group of bacteria involved in the degradation of two carboxylic acids (2-hydroxy benzoic acid and α-ketobutyric acid), which utilization was the highest in FR-treated soil.

Also in grassland soil significant changes in PLFA profiles corresponded with changes in the CLPPs. Observed increase in the mass of fatty acid biomarkers in grassland soil treated with FR on day 1 may explain increase in the substrates utilization and significant changes in pattern of CLPP in response to the same dose. In turn, increase of the mass of fatty acid biomarkers may resulted from the additional nutrients released from tetraconazole-sensitive microorganisms which died immediately after pesticide application (Wu et al. 2014). Interestingly, only application of FR tetraconazole increased substrates utilization as estimated by Biolog profiles, and stimulating effect was not observed in 10FR-treated soil. Similar effect was observed by Černohlávková et al. (2009) who found that microbial activity measured as basal respiration and substrate-induced growth was stimulated at lower application dose of fungicides mancozeb and dinocap.

Additionally, analysis of values of functional biodiversity index revealed that tetraconazole affected metabolic potential of bacterial community. Stimulating effect of tetraconazole application in the grassland soil was transient and decrease in the values of *H* and *Rs* indices were detected 28 days after soil contamination. Decreasing of the value of *H* index in response to the application of tetraconazole into the soil without history of pesticide application was observed also during whole experimental period by Zhang et al. (2014a). In opposite to the changes in the values of biodiversity indices calculated based on the DGGE data, indicated higher sensitivity of microorganism from orchard, values of functional biodiversity indices calculated for orchard soil were not significantly important. It may be explained by the fact, that some microorganisms responded differently on the application of tetraconazole. In our study the decrease in genetic diversity in orchard microbial community was not connected with the decline of the functional diversity of bacterial population. Generally, the values of Shannon index differed depending on pesticide use, type of soil or time of incubation what explain variability of the value of *H* found in various studies (Fließbach and Mäder 2004; Muñoz-Leoz et al. 2011; Wang et al. 2012; Zhang et al. 2014b; Wu et al. 2015). In our study total soil activity expressed as FDA hydrolysis was lower in orchard in comparison with grassland soil, however increase of microbial activity in respond to FR and 10FR tetraconazole was observed only in the orchard soil. Previously described decrease in mass of GP PLFAs markers and no changes in biomass of fungi may indicate toxic effect of fungicide mainly on GP bacteria. Therefore utilization of applied fungicide and/or dead bacterial cells as a source of carbon and energy by other microbial groups (Wu et al. 2014) may explain the general increase in the total microbial activity in response to tetraconazole in orchard soil. That kind of changes were not observed in soil without history of pesticide application, where main decreasing effect of tetraconazole on the total activity was observed only in soil applied with the highest dose of fungicide (10FR). Various sensitivities of microbial activity (FDA) analysis in soils with different history of pesticide application and management (agricultural and grassland soil) was observed also in response to iprodione application (Verdenelli et al. 2012). Similarly, results of the study conducted by Marshall et al. (2011) indicated that microbial community of grassland soil was relatively insensitive to benomyl application. Generally, in our study the response of microorganisms to pesticide in grassland soil was faster, than in orchard soil. This fact may be connected with higher diversity of bacterial community in grassland, which increase the probability that some fraction of microbial community response rapidly to applied pesticide.

It is expected that regular annual application of pesticide, like in orchard, may lead to accumulation of pesticides and creation of selective pressure. Bacteria fast adopt to new environmental conditions (Jacobsen and Hjelmsø 2014) and as response to the pressure, altered microbial community may appear, which is able to operate under pesticide pressure (Arbeli and Fuentes 2007). Nonetheless long-term pesticide pressure could also reduce capacity of agroecosystem to self-regulation (Barrios 2007). In the light of previous discussed results of our study, decreasing of the resilience, described as amount of time require by microbial community for recovery to predisturbance levels, in response to tetraconazole application has been observed (Mele and Crowley 2008).

## Conclusions

History of soil management has significant impact on microbial parameters and differed the response of the soil microbial community to fungicide tetraconazole. The orchard soil with a long-term history of pesticide application was characterised by lower values of the genetic diversity indices, microbial biomass and total soil activity. The values of stress indices revealed the stress condition in apple orchard soil. Moreover, obtained results indicated that orchards soil seems to be more vulnerable to the application of new fungicide in comparison with grassland soil without history of pesticide application. Application of FR and 10FR tetraconazole in the soil collected from apple orchard affecting the structure of soil microbial community and genetic and functional diversity of bacterial community and confirmed the potential of the fungicide tetraconazole to impact non-target soil microorganisms. These conclusions should be taken into account in assessing of environmental impact of studied pesticides.

## Electronic supplementary material

Below is the link to the electronic supplementary material.

Supplementary material 1 (DOCX 50 kb)

Supplementary material 2 (DOCX 46 kb)
