# Supplementary material for: Non-target impact of fungicide tetraconazole on microbial communities in soils with different agricultural management
Source: Ecotoxicology. 2016 Apr 22;25:1047–60. doi: 10.1007/s10646-016-1661-7 (PMC4921116; doi:10.1007/s10646-016-1661-7)
Supplement: Supplementary file 1 — Supplementary material 1 (DOCX 50 kb) [file 10646_2016_1661_MOESM1_ESM.docx]

Table S1. Phospholipid fatty acid (PLFA) fractions (as nmol g^-1^ of dry soil) and stress indices for orchard (O) and grassland (G) soils after treatment at FR and 10FR of tetraconazole; C – control.

| Soil | Time | Dose | Total PLFAs | Bacterial PLFAs | Gram-negative PLFAs | Gram-positive PLFAs | Actinomycetal PLFAs | Fungal PLFAs | pre/cy ratio | S/M ratio | GN/GP ratio | F/B ratio |
| --- | --- | --- | --- | --- | --- | --- | --- | --- | --- | --- | --- | --- |
| *P value* | | | *0.060* | *0.153* | *0.418* | *0.062* | *0.139* | *0.324* | *0.433* | *0.199* | ***0.044*** | *0.277* |
| O | 1 | C | 103.37±1.03 | 37.56±0.55 | 15.49±0.92 | 14.63±1.35 | 7.45±0.57 | 6.97±0.47 | 0.49±0.04 | 1.48±0.06 | 1.07±0.15^bcd^ | 0.06±0.01 |
|  |  | FR | 104.47±8.95 | 37.66±2.76 | 15.49±0.82 | 14.86±2.35 | 7.30±0.22 | 7.21±1.00 | 0.50±0.07 | 1.41±0.10 | 1.06±0.15^cd^ | 0.07±0.01 |
|  |  | 10FR | 115.20±5.92 | 39.91±1.04 | 15.53±0.57 | 16.74±0.39 | 7.65±0.31 | 7.35±0.55 | 0.52±0.04 | 1.75±0.29 | 0.93±0.01^d^ | 0.06±0.01 |
|  | 7 | C | 88.48±8.18 | 33.49±2.16 | 13.97±0.94 | 13.38±0.40 | 6.14±0.88 | 6.72±0.39 | 0.60±0.06 | 1.24±0.22 | 1.04±0.04^cd^ | 0.07±0.01 |
|  |  | FR | 92.98±7.14 | 31.99±1.85 | 13.60±1.10 | 11.85±0.87 | 6.55±0.14 | 6.79±0.46 | 0.54±0.04 | 1.58±0.51 | 1.15±0.03^bc^ | 0.07±0.01 |
|  |  | 10FR | 93.92±8.67 | 36.02±3.84 | 14.92±1.28 | 13.59±1.38 | 7.50±1.18 | 7.26±0.74 | 0.58±0.03 | 1.32±0.01 | 1.10±0.02^bc^ | 0.07±0.00 |
|  | 14 | C | 86.95±4.54 | 40.87±2.61 | 16.83±0.70 | 15.62±0.96 | 8.42±0.96 | 8.56±0.61 | 0.61±0.05 | 0.87±0.05 | 1.08±0.03^bcd^ | 0.07±0.00 |
|  |  | FR | 75.01±2.26 | 37.49±1.57 | 15.76±0.82 | 14.18±0.59 | 7.56±0.49 | 7.12±0.33 | 0.63±0.04 | 0.92±0.06 | 1.11±0.04^bc^ | 0.06±0.01 |
|  |  | 10FR | 71.40±13.93 | 35.45±5.98 | 15.19±2.75 | 13.13±2.19 | 7.12±1.20 | 7.26±1.17 | 0.58±0.06 | 0.81±0.04 | 1.16±0.10^bc^ | 0.06±0.00 |
|  | 21 | C | 70.28±2.31 | 34.66±0.73 | 14.65±0.14 | 12.82±0.61 | 7.20±0.28 | 7.40±0.06 | 0.65±0.03 | 0.95±0.07 | 1.14±0.05^bc^ | 0.07±0.01 |
|  |  | FR | 66.18±1.83 | 33.09±2.20 | 13.76±1.08 | 12.29±0.66 | 7.04±0.70 | 6.79±0.38 | 0.70±0.03 | 0.92±0.06 | 1.12±0.08^bc^ | 0.06±0.00 |
|  |  | 10FR | 60.57±5.74 | 30.21±3.18 | 12.95±1.69 | 10.64±0.91 | 6.62±0.62 | 6.58±0.98 | 0.68±0.04 | 0.91±0.08 | 1.22±0.07^b^ | 0.07±0.01 |
|  | 28 | C | 64.15±16.61 | 33.75±7.89 | 14.11±3.34 | 11.97±3.40 | 7.67±1.33 | 6.61±1.46 | 0.71±0.02 | 0.86±0.12 | 1.20±0.15^bc^ | 0.06±0.00 |
|  |  | FR | 45.02±11.71 | 24.18±6.42 | 10.88±2.45 | 7.48±2.65 | 5.82±1.41 | 5.28±1.05 | 0.37±0.01 | 0.90±0.04 | 1.50±0.18^a^ | 0.06±0.01 |
|  |  | 10FR | 57.83±4.08 | 30.39±2.16 | 13.68±0.88 | 9.87±0.88 | 6.83±0.57 | 6.39±0.60 | 0.70±0.04 | 0.88±0.06 | 1.39±0.06^a^ | 0.06±0.00 |
| *P value* | | | *0.695* | *0.447* | *0.246* | *0.823* | *0.386* | *0.333* | ***0.002*** | *0.733* | *0.158* | *0.862* |
| G | 1 | C | 87.70±8.98 | 43.02±4.95 | 19.82±2.64 | 15.28±1.56 | 7.92±0.95 | 8.74±1.10 | 0.31±0.01^h^ | 0.62±0.05 | 1.30±0.09 | 0.07±0.01 |
|  |  | FR | 105.85±6.15 | 53.79±3.54 | 25.30±1.80 | 18.39±1.49 | 10.10±0.33 | 10.71±0.68 | 0.32±0.01^h^ | 0.60±0.03 | 1.38±0.05 | 0.06±0.00 |
|  |  | 10FR | 97.73±8.75 | 49.34±3.62 | 23.59±2.06 | 16.34±1.69 | 9.41±0.27 | 10.38±1.14 | 0.31±0.00^h^ | 0.58±0.01 | 1.44±0.03 | 0.07±0.00 |
|  | 7 | C | 81.36±11.14 | 40.65±5.59 | 18.65±2.79 | 14.35±1.21 | 7.65±1.61 | 7.74±1.33 | 0.36±0.00^fg^ | 0.72±0.08 | 1.30±0.09 | 0.06±0.01 |
|  |  | FR | 87.86±0.88 | 42.78±1.06 | 19.81±0.68 | 15.04±0.93 | 7.92±0.47 | 8.02±0.40 | 0.35±0.02^g^ | 0.68±0.05 | 1.32±0.08 | 0.06±0.01 |
|  |  | 10FR | 87.88±8.13 | 44.13±5.55 | 20.52±2.04 | 15.01±2.42 | 8.59±1.26 | 8.59±0.85 | 0.38±0.01^ef^ | 0.68±0.04 | 1.38±0.11 | 0.07±0.02 |
|  | 14 | C | 78.85±10.06 | 39.02±4.53 | 18.09±1.81 | 13.56±2.09 | 7.37±1.12 | 7.50±0.93 | 0.38±0.00^def^ | 0.66±0.00 | 1.35±0.14 | 0.06±0.00 |
|  |  | FR | 81.77±11.20 | 40.78±6.16 | 18.87±2.74 | 14.54±2.51 | 7.38±0.98 | 7.70±1.24 | 0.38±0.01^cdef^ | 0.71±0.01 | 1.30±0.07 | 0.05±0.00 |
|  |  | 10FR | 80.62±4.16 | 39.36±1.45 | 18.19±0.44 | 14.15±0.79 | 7.02±0.22 | 7.58±0.38 | 0.39±0.01^cde^ | 0.69±0.04 | 1.29±0.04 | 0.06±0.00 |
|  | 21 | C | 87.98±8.28 | 41.98±4.59 | 19.28±2.59 | 14.28±2.25 | 8.42±0.10 | 7.45±0.67 | 0.41±0.02^b^ | 0.74±0.01 | 1.36±0.10 | 0.05±0.00 |
|  |  | FR | 78.13±29.22 | 34.71±14.62 | 15.32±6.31 | 12.64±4.98 | 6.75±3.39 | 6.16±1.94 | 0.47±0.02^a^ | 0.79±0.11 | 1.20±0.03 | 0.06±0.01 |
|  |  | 10FR | 87.95±3.64 | 40.09±1.94 | 18.04±0.82 | 14.33±0.49 | 7.73±0.74 | 7.19±0.46 | 0.42±0.02^b^ | 0.73±0.11 | 1.26±0.02 | 0.05±0.00 |
|  | 28 | C | 69.01±4.69 | 35.48±3.06 | 16.02±1.41 | 13.15±1.24 | 6.31±0.51 | 7.34±0.77 | 0.40±0.01^bcd^ | 0.65±0.02 | 1.22±0.03 | 0.07±0.03 |
|  |  | FR | 69.09±7.38 | 36.12±4.09 | 16.32±1.73 | 13.25±1.63 | 6.55±0.80 | 6.95±0.51 | 0.41±0.01^b^ | 0.63±0.03 | 1.23±0.06 | 0.06±0.00 |
|  |  | 10FR | 72.87±11.01 | 37.76±6.04 | 17.34±2.48 | 13.48±2.70 | 6.94±0.89 | 7.27±1.08 | 0.40±0.01^bc^ | 0.63±0.01 | 1.30±0.09 | 0.06±0.01 |

The data presented are the means and standard deviations of three replicates. Different letters indicate significant differences (*P<*0.05, LSD test), considering the effects of the pesticide dosage and time for each soil.
