# Supplementary material for: Non-target impact of fungicide tetraconazole on microbial communities in soils with different agricultural management
Source: Ecotoxicology. 2016 Apr 22;25:1047–60. doi: 10.1007/s10646-016-1661-7 (PMC4921116; doi:10.1007/s10646-016-1661-7)
Supplement: Supplementary file 2 — Supplementary material 2 (DOCX 46 kb) [file 10646_2016_1661_MOESM2_ESM.docx]

Table S2. PCA factor loadings in Biolog data.

| Biolog-Eco Plate substrates | | Orchard | |  | Grassland | |
| --- | --- | --- | --- | --- | --- | --- |
|  |  | PC1 | PC2 |  | PC1 | PC2 |
| amines amides | phenylethylamine | -0.61 | 0.09 |  | -0.62 | -0.23 |
|  | putrescine | -0.76 | 0.09 |  | -0.19 | 0.47 |
| amino acids | glycyl-L-glutamic acid | -0.45 | -0.53 |  | -0.55 | 0.52 |
|  | L-arginine | -0.66 | -0.23 |  | -0.83 | -0.24 |
|  | L-asparagine | -0.72 | -0.32 |  | -0.48 | 0.13 |
|  | L-phenylalanine | -0.46 | -0.06 |  | -0.29 | -0.47 |
|  | L-serine | -0.78 | -0.15 |  | -0.71 | -0.07 |
|  | L-threonine | -0.25 | 0.34 |  | 0.03 | 0.44 |
| carbohydrates | α-D-lactose | -0.38 | 0.86 |  | -0.81 | 0.00 |
|  | ß-methyl-D-glucoside | -0.76 | 0.31 |  | -0.78 | -0.43 |
|  | D-cellobiose | -0.88 | 0.31 |  | -0.77 | -0.48 |
|  | D-mannitol | -0.71 | 0.16 |  | -0.9 | -0.17 |
|  | D-xylose | -0.73 | 0.48 |  | -0.88 | -0.04 |
|  | i-erythritol | 0.16 | -0.29 |  | -0.42 | 0.55 |
|  | N-acetyl-D-glucosamine | -0.67 | -0.08 |  | -0.51 | -0.48 |
| carboxylic acids | γ-hydroxybutyric acid | -0.29 | -0.08 |  | 0.04 | -0.11 |
|  | α-ketobutyric acid | 0.22 | 0.62 |  | -0.24 | 0.77 |
|  | 2-hydroxy benzoic acid | 0.17 | 0.71 |  | 0.11 | 0.28 |
|  | 4-hydroxy benzoic acid | -0.81 | 0.03 |  | -0.86 | 0.02 |
|  | D-galactonic acid α-lactone | -0.86 | 0.00 |  | -0.71 | 0.13 |
|  | D-galacturonic acid | -0.72 | -0.34 |  | -0.69 | 0.31 |
|  | D-glucosaminic acid | -0.53 | -0.43 |  | -0.59 | 0.06 |
|  | D-malic acid | -0.63 | -0.18 |  | -0.39 | -0.26 |
|  | itaconic acid | -0.82 | 0.22 |  | -0.48 | -0.29 |
| miscellaneous | D,L-α-glycerol phosphate | -0.39 | 0.13 |  | -0.34 | 0.65 |
|  | glucose-1-phosphate | -0.72 | 0.50 |  | -0.65 | -0.14 |
|  | pyruvic acid methyl ester | -0.64 | -0.51 |  | -0.3 | 0.07 |
| polymers | α-cyclodextrin | -0.33 | -0.49 |  | -0.57 | 0.20 |
|  | glycogen | -0.59 | 0.25 |  | -0.63 | -0.18 |
|  | Tween 40 | -0.87 | -0.13 |  | -0.76 | 0.27 |
|  | Tween 80 | -0.69 | -0.01 |  | -0.84 | 0.46 |
